# Development and evaluation of a new survey instrument to measure the quality of colorectal cancer screening decisions

**DOI:** 10.1186/1472-6947-14-72

**Published:** 2014-08-20

**Authors:** Karen R Sepucha, Sandra Feibelmann, Carol Cosenza, Carrie A Levin, Michael Pignone

**Affiliations:** 1 Division of General Internal Medicine, Health Decision Sciences Center, Massachusetts General Hospital, 50 Staniford Street, 8th Floor, Boston, MA 02114, USA; 2 Harvard Medical School, Boston, MA 02115, USA; 3 Center for Survey Research, University of Massachusetts Boston, 100 Morrissey Boulevard, Boston, MA 02125, USA; 4 Informed Medical Decisions Foundation, 40 Court Street, 3rd Floor, Boston, MA 02108, USA; 5 UNC Division of General Internal Medicine and UNC Institute for Healthcare Quality Improvement, University of North Carolina, 5045 Old Clinic Building UNC Hospital, Chapel Hill, NC 27599-7110, USA

**Keywords:** Colon cancer screening, Decision quality, Shared decision making, Quality measurement, Survey research, Psychometrics

## Abstract

**Background:**

Guidelines for colorectal cancer screening recommend that patients be informed about options and be able to select preferred method of screening; however, there are no existing measures available to assess whether this happens.

**Methods:**

Colorectal Cancer Screening Decision Quality Instrument (CRC-DQI) includes knowledge items and patients' goals and concerns. Items were generated through literature review and qualitative work with patients and providers. Hypotheses relating to the acceptability, feasibility, discriminant validity and retest reliability of the survey were examined using data from three studies: (1) 2X2 randomized study of participants recruited online, (2) cross-sectional sample of patients recruited in community health clinics, and (3) cross-sectional sample of providers recruited from American Medical Association Master file.

**Results:**

338 participants were recruited online, 94 participants were recruited from community health centers, and 115 physicians were recruited. The CRC-DQI was feasible and acceptable with low missing data and high response rates for both online and paper-based administrations. The knowledge score was able to discriminate between those who had seen a decision aid or not (84% vs. 64%, p < 0.001) and between providers, online patients and clinic patients (89% vs. 74% vs. 41%, p < 0.001 for all comparisons). The knowledge score and most of the goals had adequate retest reliability. About half of the participants received a test that matched their goals (47% and 51% in online and clinic samples respectively). Many respondents who had never been screened had goals that indicated a preference for colonoscopy. A minority of respondents in the online (21%) and in clinic (2%) samples were both well informed and received a test that matched their goals.

**Conclusions:**

The CRC-DQI demonstrated good psychometric properties in diverse samples, and across different modes of administration. Few respondents made high quality decisions about colon cancer screening.

## Background

Clinical guidelines strongly recommend that adults age 50–75 be screened for colorectal cancer (CRC); however, guidelines also recognize that there are multiple screening options available [1]. A high quality decision in situations where there is more than one reasonable option requires ensuring that patients are informed, and that the choice of test reflects patients’ goals and concerns [2,3].

Several studies have examined the extent to which patients are informed and engaged in the decision about CRC screening. Patients have been shown to have significant knowledge gaps about screening, and lack understanding of common terms relating to colon cancer [4-6]. Studies have also identified several factors that are important to patients when considering what kind of test to have, such as reducing chance of dying of colon cancer, the invasiveness of the test, and test preparation [5,7]. Each of these studies used different measures of knowledge and different methods of assessing patients’ goals. There is a need for a comprehensive measure of decision quality that can assess whether patients are informed and receive screening tests that match their goals.

Sepucha and colleagues have developed and tested decision quality instruments that assess patients’ knowledge, and the extent to which treatments match their goals for patients facing surgical decisions, including breast cancer surgery [8-10]. A similar process was used to develop and evaluate a survey instrument to measure the quality of CRC screening decisions. This manuscript describes the development process and results of three studies that evaluated the psychometric properties (e.g. reliability, validity, and precision) and clinical sensibility (e.g. interpretability, acceptability, and feasibility) of the new survey instrument [11].

## Methods

### Item development

The Colorectal Cancer Screening Decision Quality Instrument (CRC-DQI) development process followed principles of survey research methods [12,13]. Nine candidate facts and thirteen goals were identified through a review of the clinical evidence and reports from focus groups of patients. The facts and goals were then rated on importance, accuracy and completeness by a convenience sample of patients (n = 27) and clinical experts (n = 16) including primary care physicians, nurses and gastroenterologists. Based on the ratings, new content was added (e.g. covering how often the tests need to be done, which tests require sedation) and some content was removed (e.g. quantitative estimate of disease free survival benefit from screening). Then, experts in survey research drafted questions to cover the content and conducted cognitive interviews with men and women (n = 6) to evaluate the items. Revisions to the items, responses and formatting of the survey were made to improve acceptability and comprehension. The resulting instrument was then tested in three samples.

### Study sample and procedures

#### *Study 1: online sample*

Patients were recruited through Craig’s List Internet ads in 8 U.S. cities. All respondents were screened for eligibility by a member of the study staff. Eligible respondents were adults 35–70 years of age who did not have a prior diagnosis of colon cancer. Younger participants (aged 35–49) were included in order to ensure a range of knowledge scores and adequate sample size for comparisons for the knowledge items across the different modes of administration.

Eligible participants were randomized in 2×2 design to (1) receive a decision aid or not and (2) complete the survey online or by mail. The decision aid, *Colon Cancer Screening: Deciding What’s Right For You,* is an 31 minute DVD and booklet produced by Informed Medical Decisions Foundation and Health Dialog ©2010. The initial packet included a cover letter, decision aid (if appropriate), survey (if appropriate) and incentive. Participants randomized to complete the survey online received an email with a link to the survey about 1 week after the cover letter. Non-respondents received a reminder (via email or mail) two weeks later. A subset of responders received a retest packet four weeks later (delivered in the same mode, email or mail, as the initial survey). A small incentive was provided with each survey (valued at $30 US for decision aid group, $20 US for no-decision aid group, $20 US for retest).

The study protocol for the online patient survey was approved by the Partners Human Research Committee, UMass Boston Institutional Review Board and University of Virginia Insitutional Review Board for Health Services Research. Consent was implied by the voluntary completion of the survey instruments.

#### *Study 2: clinic sample*

The online patient sample had very high education levels and limited diversity, so we recruited a sample of patients aged 50 and older from community health centers in order to examine the generalizability of the results. Patients and family members in the clinics’ waiting rooms were approached by trained staff and asked if they wanted to participate in an interview about colorectal cancer screening. Subjects did not need to have any prior experience with screening. If participants consented, they were brought to a separate room to complete a 20 minute interview followed by the written questionnaire. Staff members were trained to administer the written survey orally if the participant asked for help. Subjects were compensated $20 for participating in the interview and completing the questionnaire. The protocol was approved by the Institutional Review Boards at all participating sites.

#### *Study 3: provider survey*

We identified primary care physicians and specialists through the American Medical Association Master File from 17 cities in the United States (including the 8 cities where patients were recruited for the online study). For the colon cancer screening sample, we selected 200 physicians across internal medicine (n = 100) and gastroenterology (n = 100). Each provider was mailed a study packet with a $20 US cash incentive. A phone reminder was made at two weeks and a mailed reminder was sent at four weeks. The study protocol for the providers was approved by the Institutional Review Board and provider consent was implied by the voluntary completion of the survey.

### Measures

Patients completed demographics, screening history and the following measures.

*CRC-DQI (Colorectal Cancer Screening Decision Quality Instrument).* 16 multiple choice knowledge items and 10 goals and concerns rated on an importance scale from 0 (not at all important) to 10 (extremely important). The items used in the field test are available as Additional file 1.

*Top Three Goals and Concerns:* Patients indicated the top three goals and concerns that were most important to their decision.

*Involvement in Decision Making:* assessed with two items (1) who made the decision about testing and (2) how much they were involved in the decision.

Providers completed the full set of CRC-DQI knowledge items and were asked to rate the knowledge items overall and individually for how well they covered information that is essential for patients to know.

### Statistical Analysis

*Sample Size:* 150 participants in each group would allow us to detect a clinically meaningful difference in knowledge between decision aid and control groups of 10% with 90% power (assuming common standard deviation of 20%) and a difference in mode (online vs. mail) on response rates and on missing data of 2% (assuming common standard deviation of 0.5%) with 80% power.

*Item Retention and Deletion*: A group of experts in survey research, decision sciences and clinical experts examined responses for issues such as difficulty (e.g., too easy or too hard), problematic format (e.g., multiple responses checked off when only one was expected), redundancy (high inter-item correlation), and floor or ceiling effects (responses bunched at bottom or top of the scale). Problematic items were deleted or recommended for revision.

*DQI- Knowledge Score:* Each correct response received one point. Missing responses were considered incorrect and a total knowledge score was calculated for those who completed at least half of the items. A total knowledge score was standardized by dividing the number of correct responses by the number of items, resulting in scores from 0% to 100%.

*DQI-Concordance Score:* Respondents aged 50 and older who had a screening test were included in these analyses and were categorized by their screening history into ‘colonoscopy’ and ‘other test’ groups. Patients who had never had a screening test were not included in the regression model and for the purposes of the concordance score, they were not considered to have had a test that matched their goals. A logistic regression model, with testing group (colonoscopy vs. other test) as the dependent variable and the goals as independent variables, generated a predicted probability of having a colonoscopy. Patients with a model predicted probability of colonoscopy >0.5 and who had a colonoscopy or those with a predicted probability of colonoscopy ≤0.5 and who had some other test, were classified as a “match.” This yielded a summary concordance score that indicated the percentage of patients whose screening decisions “matched” their goals. Higher concordance scores indicate that more participants received screening tests that matched their goals.

*Decision Quality Indicator*: A binary variable was created that was “1” for patients who were both well-informed (i.e. knowledge score at or above the mean knowledge score of the decision aid group) and had a test that matched their goals, indicating high decision quality, and “0” otherwise.

*Acceptability and Feasibility:* Acceptability was examined using the response rates. Feasibility was examined using rates of missing data.

*Reliability:* Test-retest reliability was assessed by calculating the intraclass correlation coefficient (ICC) for the total knowledge score and for the individual goals and concerns. The target was to exceed 0.7 [14]. Internal consistency for the knowledge score was not calculated as the set of knowledge items is not a measure of one underlying construct.

There is no gold standard for measuring knowledge or concordance so the following hypotheses were used to examine the validity of the scores:

*Discriminant Validity:* A key feature of a knowledge test is that it can discriminate among those with different levels of knowledge. Two hypotheses were examined (a) mean knowledge scores would be higher for providers than patients (two sample *t*-test) and (b) the decision aid group would have higher knowledge than the control group (two sample *t*-test).

*Construct Validity:* The hypothesis that patients who are more involved in decision making should be more likely to have high decision quality was examined using Fisher’s exact test to compare the percentages across groups.

*Mode Effect:* Differences in response rates and rates of missing items by mode (online versus mail) were compared.

### Brief version

A short version of the instrument, with five knowledge items, was created and evaluated for reproducibility, retest reliability and discriminant validity.

Analyses were conducted using PASW Statistics 18.0.

## Results

### Response rates and sample

For the online sample, 338/367 (92%) participants responded to the initial survey and 71/84 (84.5%) responded to the retest survey. The decision aid and no decision aid arms were balanced on all demographic characteristics. For the clinic sample, 94 participants were enrolled (data regarding response rate was not available). Participant characteristics for these two samples are in Table 1. For the provider sample, 115/193 (59.6%) responded and their characteristics are summarized in Table 2. Provider non responders were slightly younger than responders (51 vs. 53 years old, p = 0.04), but did not differ by gender or specialty.

**Table 1:** Patient demographics for the online and in-clinic samples

		**Online**		**Clinic sample**
	**Sample**			
	**Total**	**DA**	**No DA**	**Total**
Characteristic*, **	**N = 338**	**N = 163**	**N = 175**	**N = 94**
**Female (%)**	229 (68%)	107 (66%)	122 (70%)	38 (40%)
**Age mean (SD)**	48.9 (9.0)	49.0 (9.1)	48.5 (9.2)	59.6 (8.3)
**Hispanic (%)**	34 (10%)	15 (9%)	19 (11%)	0 (0%)
**Race (%)**				
White	289 (85.5%)	140 (86%)	149 (85%)	47 (50%)
Black	31 (9%)	17 (10.4%)	14 (8%)	44 (47%)
Other race	19 (6%)	8 (5%)	11 (6%)	3 (3%)
**Education (%)**				
≥ College graduate	193 (57%)	97 (59.5%)	96 (55%)	9 (10%)
Some college	114 (34%)	50 (31%)	64 (37%)	25 (27%)
High school or less	31 (9%)	16 (10%)	15 (9%)	59 (63%)
**Screening History (For respondents 50 and older)**	N = 150	N = 80	N = 68	N = 94
Never tested	53 (35%)	34 (43%)	19 (27%)	29 (31%)
Colonscopy	68 (45%)	35 (44%)	33 (47%)	54 (57%)
Other test only	29 (19%)	11 (14%)	18 (26%)	8 (9%)

DA = Decision aid.

*There were no significant differences on any of these factors between DA and no DA arms, p > 0.05.

**Percentages might not add up 100% due to rounding or missing data.

**Table 2:** Provider demographics

	**N = 115**
**Gender (Male)**	85 (74%)
**Age mean (SD)**	53 (9)
**# of Years in Practice**	22 (SD 9.6)
Mean (SD)	
**Annual patient volume Median (IQ)**	1,000 (Q1: 450; Q3: 1,100)
**Hispanic (%)**	6 (5%)
**Race (%)**	
White	78 (68%)
Black	6 (5%)
Asian	25 (22%)
Other race	3 (3%)
**Professional Training (%)**	
Primary care physician	56 (49%)
Gastroenterologist	58 (50%)
Nurse practitioner	1 (1%)

### Item retention and deletion

Six knowledge items were identified as being too easy (i.e., patient scores were >85%). Four of them were removed because the provider importance scores were also low and two were revised to be included in the future version of the instrument. Across both patient samples, total knowledge scores ranged from 10-100% with no evidence of a floor or ceiling effect.

Responses for the goals ranged across the entire set of options, though three of the goals had evidence of a ceiling effect. Many respondents selected 10 out of 10 for “to know whether or not you have colon cancer” (64%), “to try to find colon cancer or polyps early” (57%), and “to avoid a test that can cause bleeding or a tear in the colon” (56%). These three items were kept in for analyses because they also had the highest percentage of patients who indicated that each goal was one of their top three issues. Two goals were eliminated as few patients included them as one of their top three goals: “avoid a test where you have to drink a liquid before the test to clean out your colon (6% selected this item), and “avoid a test that requires you to handle your stool” (only 5% selected this item).

The remaining analyses were conducted using a reduced set of 10 knowledge items and the 8 goals and concerns.

### Acceptability and feasibility

The survey had a high response rate 338/367 (92%) and few missing items (1.3% for knowledge items and 0.7% for goals on average) for the online patient sample. Missing items did vary by mode of administration for knowledge (2.5% vs. 0.2%, p < 0.001 for paper and online versions respectively) but did not vary by mode for the goals. There were more missing items in the clinic sample, but overall the amount was still low, 3.2% (range 1.2-7.4%) for knowledge items and 4.0% (range 1.1 – 5.3%) for goal items. None of the online respondents and only one respondent in the clinic sample was missing more than half of the knowledge items and did not receive a total knowledge score.

### Reliability

The knowledge score had retest reliability ICC = 0.67 (95% CI 0.47, 0.79). The retest reliability of three goals was strong “find colon cancer or polyps early” (ICC = 0.87), “to know whether or not you have colon cancer” (ICC = 0.85), and “avoid test where a tube is put into your rectum to look at the colon” (ICC = 0.74). Three others were modest “avoid test that is painful” (ICC = 0.68), “to avoid a test that can cause bleeding or a tear in the colon” (ICC = 0.68), and “choose test that doesn’t cost you a lot of money” (ICC = 0.61). Two had low retest reliability “chose a test that does not need to be done every year” (ICC = 0.47) and “chose a test where you take medicine before the test that makes you sleepy” (ICC = 0.55).

### Knowledge score

Participants in the decision aid (DA) group had higher knowledge scores than those in the control, (84% vs. 64%, p < 0.001). This difference remained significant when the sample was restricted to the population aged 50 and older (e.g. estimated mean difference 17.5% (95%CI 12.1%, 22.9%), p = 0.001 in the DA and non DA groups). Providers also had significantly higher knowledge scores than the online sample (88.7% vs. 73.8%, p < 0.001). Table 3 shows scores on the individual knowledge items across the different samples. The clinic sample, none of whom had viewed a decision aid, had significantly lower mean knowledge score than the online sample (40.6% vs. 73.8%, p <0.001).

**Table 3:** Correct responses to knowledge items for each sample

	**Online**		**Providers**	**Clinic sample**
	**Sample**			
**Question (correct answer)**	**DA**	**No DA**		
	**N = 163**	**N = 175**	**N = 115 (%)**	**N = 94 (%)**
1. At what age do doctors usually recommend people start getting regular tests for colon cancer? (50)*	150 (92%)	126 (72%)	114 (99%)	50 (53%)
2. Out of every 100 people about how many will get colon cancer some time in their lives? (6)*	109 (67)	63 (36%)	66 (57%)	6 (6%)
3. Does having a colon cancer test result that is not normal always mean that a person has colon cancer? (No)	155 (95%)	168 (96%)	113 (98%)	81 (86%)
4. How often do serious problems, such as serious bleeding or a tear in the colon, happen as a result of a colonoscopy? (Rarely)*	151 (93%)	125 (71%)	109 (95%)	55 (59%)
5. For a person with an average risk for colon cancer, which test do doctors recommend be done every year? (Stool blood test)*	140 (86%)	96 (55%)	112 (97%)	28 (30%)
6. For a person with an average risk for colon cancer, which test do doctors recommend be done every 10 years? (Colonoscopy)*	147 (90%)	115 (66%)	111 (97%)	48 (51%)
7. How does regular testing for colon cancer change the chances that a person will die from colon cancer? (Decreases chance)	156 (96%)	160 (91%)	113 (98%)	70 (75%)
8. Which colon cancer test is least likely to miss a cancer? (Colonoscopy)*	92 (56%)	72 (41%)	100 (87%)	21 (22%)
9. If the results of a colon cancer test are normal, is it possible that a person could still have colon cancer? (Yes)	136 (83%)	148 (85%)	110 (96%)	71 (76%)
10. Out of every 100 people about how many will die of colon cancer? (3)*	133 (82%)	53 (30)	73 (64%)	12 (13%)

DA = decision aid; *p < 0.05 for DA vs. no DA.

The majority of providers (81%) felt that the set of knowledge items covered the key facts extremely or very well, supporting content validity. The provider ratings of whether the individual items were “essential” varied from 17% for “Out of every 100 people, about how many will die from colon cancer?” to 69% for “At what age do doctors usually recommend people start getting regular tests for colon cancer.”

### DQI-concordance score

**Figure 1:**
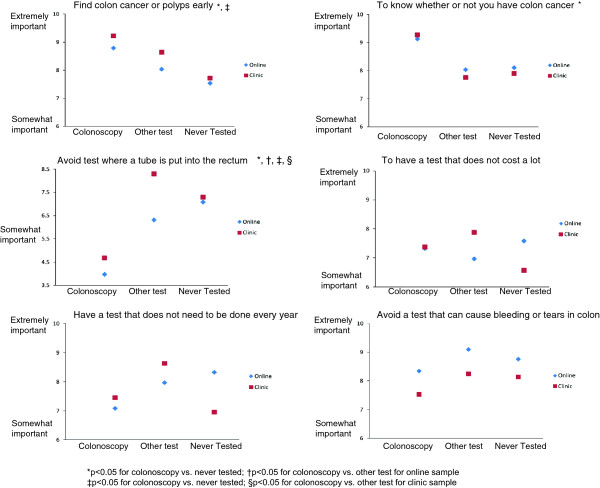
Mean importance scores for selected goals for online and clinic samples based on screening status.

Figure 1 contains the plots of the mean importance scores on selected goals for patients who reported having had a colonoscopy, some other screening test and never being screened. Three goals, desire to “find colon cancer or polyps early,” “know whether or not you have cancer,” and “avoid a test where a tube is put into your rectum to look at the colon,” discriminated significantly between those who had colonoscopy and those who did not in the online sample (see Figure 1). In multivariable analysis, two of these goals were associated with having colonoscopy versus some other test (see Table 4). Patients who felt it was important to “know whether or not you had colon cancer” were more likely to have a colonoscopy and patients who felt it was important to “avoid a test where a tube is put into rectum to look at the colon” were less likely to have a colonoscopy. The concordance score, or number of participants in the online sample who received a screening test that matched that predicted by their stated goals was 47.3% (71/150).

**Table 4:** Multivariate analyses of factors associated with getting colonoscopy versus other screening test from online sample

**Factor**	**Odds ratio of Colonoscopy (95%CI)**
**To know whether or not you have colon cancer**	**1.83 (1.16, 2.90)**
**To avoid a test where a tube is put into your rectum**	**0.70 (0.56, 0.88)**
To avoid a test that can cause bleeding or a tear in the colon	0.84 (0.58, 1.21)
To try to find colon cancer or polyps early	0.74 (0.48, 1.14)
To choose a test that does not need to be done every year	1.15 (0.94, 1.40)
To choose a test that doesn’t cost you a lot of money	0.95 (0.79, 1.14)
To avoid a test that may be painful	1.19 (0.91, 1.55)

Bolded factors were significant in the multivariate model.

The fifty-two respondents who were 50 or older and who had never been screened in the online sample were not considered to have received a test that matched their goals. The majority of them (57%) had goals that suggested a preference for colonoscopy and 43% had goals that suggested some other screening test would be the best fit. In the clinic sample, (35/74) 47.3% had a screening test that matched their goals. About half the clinic participants who had never been screened (53.8%) had goals that suggested a preference for colonoscopy.

### Decision quality indicator

In the online sample, 32/151 (21.2%) met the definition of high decision quality, (i.e. knowledge score at or above 84%, the mean of the decision aid group, and had a test that matched their goals). Participants with high decision quality were *less* likely to say that the doctor made the decision compared to those who did not meet criteria for high decision quality, but the difference was not significant (29.7% vs. 38.9%, p = 0.25 for the online sample). For the clinic sample, only two repondents met the threshold for being considered informed, and as a result, only 3% of respondents met our definition of high decision quality.

### Brief version

A shorter, five item version of the knowledge test had retest reliability (ICC = 0.54) and reproducibility to the total knowledge score (Pearson R = 0.89). The knowledge scores using the brief version also discriminated between the DA group and the control (85.4% vs. 58.9%, p <0.001) and between providers and patients in the online sample (82.1% vs. 74.3%, p = 0.008).

## Discussion

The CRC-DQI is a new survey that examines the extent to which patients are informed and receive tests that match their goals. High acceptability and feasibility of the instrument is evidenced by the high response rate and the low number of missing responses in diverse patient samples. The survey instrument is acceptable and feasible when administered online, by mail or in person.

The knowledge score was able to discriminate among those who were more or less informed, including providers and patients, and those who have seen a decision aid or not. The participants in the decision aid group and virtually all providers had a good understanding of the frequency of screening for the different tests and the likelihood of serious side effects with colonoscopy. Participants in the decision aid arm were also more likely to have realistic expectations for the incidence and lethality of colon cancer, significantly more accurate than providers (p < 0.001). The overall magnitude of the improvement in knowledge for the decision aid group compared to the control was more than that seen in the Cochrane Collaborative systematic review of decision aids [15].

Few patients from the clinic sample, which was a more diverse and underserved sample, were able to answer the knowledge items correctly. Other studies have found knowledge gaps about colon cancer screening, particularly in underserved populations [16,17]. Decision aids, like the one used in the online sample, have been shown to increase knowledge and intention to screen, even in underserved samples [15,18]. It may be more important to distribute these types of tools to underserved patients to ensure informed decisions.

There are several screening options available to patients. Three goals appeared to have strongest association with screening choice: the desire to find colon cancer early and to know whether you have cancer appeared to be traded off with the desire to avoid invasive tests. These three goals had good retest reliability and were able to discriminate among those who had colonoscopy versus some other test. To support shared decision making conversations around colon cancer testing, it would be important for providers to assess, at a minimum, how patients feel about these three issues.

In this study, those who did not get screened were categorized as not having made a “concordant” decision. Guidelines clearly support some sort of colon cancer screening for men and women age 50–75 [1]. It is certainly possibly that an individual may consider the potential benefits and harms of colon cancer screening and make an informed decision not to pursue screening. However, about half of the respondents who did not report any screening test for each sample, had goals that indicated a preference for colonoscopy. The other half were not significantly different from those who had some other test.

A small percentage in each group met both of the criteria for a high quality decision, suggesting considerable room for improvement across all samples. The level of decision quality did not vary by the type of test (colonoscopy vs. other test). The results from this study did not find an association between self reported participation in the decision and decision quality. Studies have suggested that engaging patients though the use of decision aids or offering them a choice of screening options may increase screening rates [19-21]. It would be important for future studies to examine whether higher decision quality leads to higher screening rates.

There are several limitations to the studies that should be noted. The retrospective surveys are subject to recall bias and the respondents’ knowledge and goals may be different if assessed closer to the time of the actual decision. The sample recruited online was well educated, had Internet access and may not generalize to the wider population. The clinic sample had much more diversity with respect to race, socioeconomic variables and education; however, it was limited to English speaking respondents and we did not have information on clinic non responders. Further, some of the differences between the online and clinic samples may be due to differences in mode of administration (e.g. those completing the survey online may have looked up information regarding the knowledge items whereas those completing it in clinic did not have that opportunity). Despite these limitations, the studies do provide considerable data to evaluate the properties of the survey instrument and its use in different populations, and with different modes of administration.

## Conclusions

The CRC-DQI is a new survey instrument that has demonstrated strong content validity, discriminant validity and moderate retest reliability. As guidelines increasingly emphasize the importance of informing patients and offering them a choice of screening tests for colon cancer, the CRC-DQI provides a validated means of measuring whether that occurs. The survey may be administered online, by mail, or in person, to evaluate the extent to which colon cancer screening decisions are informed and match patients’ goals.

## Competing interests

Dr. Sepucha receives salary and research support from the Informed Medical Decisions Foundation. Dr. Levin receives salary support as Research Director for the Informed Medical Decisions Foundation, a not-for-profit (501 (c) 3) private foundation (http://www.informedmedicaldecisions.org). The funding agreements ensured the authors’ independence in designing the study, interpreting the data, writing, and publishing the report.

The Foundation develops content for patient education programs. The Foundation has an arrangement with a for-profit company, Health Dialog, to co-produce these programs. The programs are used as part of the decision support and disease management services Health Dialog provides to consumers through health care organizations and employers.

## Authors’ contributions

All authors contributed substantially to one or more of the studies including (1) the conception and design of patient study (KRS, CC, CL) and provider study (KRS, CC, CL), acquisition of data (KRS, SF, CC), or analysis and interpretation of data (all authors) (2) drafting the article or revising it critically for important intellectual content (all authors) (3) final approval of the version to be submitted (all authors). The corresponding author, KRS (ksepucha@partners.org), is responsible for the integrity of the work as a whole.

## Pre-publication history

The pre-publication history for this paper can be accessed here:

http://www.biomedcentral.com/1472-6947/14/72/prepub

## Supplementary Material

Additional file 1 CRC-DQI field test items. Click here for file
