# Supplementary material for: Development and evaluation of a new survey instrument to measure the quality of colorectal cancer screening decisions
Source: BMC Med Inform Decis Mak. 2014 Aug 20;14:72. doi: 10.1186/1472-6947-14-72 (PMC4147095; doi:10.1186/1472-6947-14-72)
Supplement: Additional file 1 — CRC-DQI field test items. [file 1472-6947-14-72-S1.pdf]

Testing for Colon Cancer

SURVEY INSTRUCTIONS

- Your participation in this study is voluntary. If you come across a question you would rather not answer, feel free to skip it and go on to the next question.
- Your answers are confidential. No information will be presented or published in any way that would permit identification of any individual. Your name and answers will not be shared with anyone other than the researchers.
- Answer all the questions by checking the box next to your answer.

Things to consider

Start here: People consider many things when thinking about getting tested for colon cancer. We would like to know what is important to <u>you</u>. Please rate each of the items below using any number from 0 to 10, where 0 is not at all important, 5 is somewhat important, and 10 is extremely important.

A1. When you think about whether or not you want to have a test for colon cancer, how important is it to you …

	Not at all important to me					Somewhat important to me					Extremely important to me
a. …to try to find colon cancer or polyps early?	0 ☐	1 ☐	2 ☐	3 ☐	4 ☐	5 ☐	6 ☐	7 ☐	8 ☐	9 ☐	10 ☐
b. …to know whether or not you have colon cancer?	0 ☐	1 ☐	2 ☐	3 ☐	4 ☐	5 ☐	6 ☐	7 ☐	8 ☐	9 ☐	10 ☐

A2. When you think about which kind of colon cancer test to have, how important is it to you …

	Not at all important to me					Somewhat important to me					Extremely important to me
a. …to choose a test that does not need to be done every year?	0 ☐	1 ☐	2 ☐	3 ☐	4 ☐	5 ☐	6 ☐	7 ☐	8 ☐	9 ☐	10 ☐
b. …to choose a test where you take medicine before the test that makes you sleepy?	0 ☐	1 ☐	2 ☐	3 ☐	4 ☐	5 ☐	6 ☐	7 ☐	8 ☐	9 ☐	10 ☐
c. ... to choose a test that doesn't cost you a lot of	0 ☐	1 ☐	2 ☐	3 ☐	4 ☐	5 ☐	6 ☐	7 ☐	8 ☐	9 ☐	10 ☐

A3. When people think about which kind of colon cancer test to have, sometimes there are things they specifically want to **avoid**. What number would you use to rate how important it is to you …

	0 Not at all important to me	1	2	3	4	5 Somewhat important to me	6	7	8	9	10 Extremely important to me
a. …to **avoid** a test that requires you to handle your stool?	0 ☐	1 ☐	2 ☐	3 ☐	4 ☐	5 ☐	6 ☐	7 ☐	8 ☐	9 ☐	10 ☐
b. …to **avoid** a test that may be painful?	0 ☐	1 ☐	2 ☐	3 ☐	4 ☐	5 ☐	6 ☐	7 ☐	8 ☐	9 ☐	10 ☐
c. …to **avoid** a test where a tube is put into your rectum to look at the colon?	0 ☐	1 ☐	2 ☐	3 ☐	4 ☐	5 ☐	6 ☐	7 ☐	8 ☐	9 ☐	10 ☐
d. …to **avoid** a test that can cause bleeding or a tear in the colon?	0 ☐	1 ☐	2 ☐	3 ☐	4 ☐	5 ☐	6 ☐	7 ☐	8 ☐	9 ☐	10 ☐
e. …to **avoid** a test where you have to drink a liquid before the test to clean out the colon?	0 ☐	1 ☐	2 ☐	3 ☐	4 ☐	5 ☐	6 ☐	7 ☐	8 ☐	9 ☐	10 ☐

Facts about Colon Cancer Tests

Start here: These questions ask about your understanding of colon cancer tests. The correct answers are based on medical research and practice. Please do your best to answer each question, even if you did not have the test discussed in the question.

C1. For each of the following, mark whether it is a way to test for colon cancer.

a.	Testing a urine sample	☐ Yes	☐ No
b.	Testing the stool for blood	☐ Yes	☐ No
c.	Looking inside the colon by putting a tube in the rectum	☐ Yes	☐ No
d.	Testing blood taken from the arm	☐ Yes	☐ No

C2. At what age do doctors usually recommend people start getting regular tests for colon cancer?

☐ 30
☐ 40
☐ 50
☐ 60

C3. How do most colon cancers start?

☐ As a tear in the colon
☐ As a polyp in the colon
☐ As a result of constipation
☐ As a hemorrhoid

C4. For each of the following, mark whether or not it can increase the chance of a person getting colon cancer.

a.	Being over age 50	☐ Yes	☐ No
b.	Having a history of inflammatory bowel disease	☐ Yes	☐ No
c.	Having heart disease	☐ Yes	☐ No
d.	Having a family history of colon cancer	☐ Yes	☐ No

C5. You may not know the exact number, but please take your best guess. Out of every 100 people, about how many will **get** colon cancer some time in their lives? Please mark the number that you think is closest to the correct answer.

- ☐ 2
- ☐ 6
- ☐ 14
- ☐ 24
- ☐ 43

C6. Before some tests for colon cancer, people may be required to clean out their colon by drinking a lot of liquid that makes them move their bowels a lot. For each of the following colon cancer tests, mark whether or not it usually requires people to clean out their colon before the test.

a.	Stool Blood Test	☐ Yes	☐ No
b.	Colonoscopy	☐ Yes	☐ No
c.	Sigmoidoscopy	☐ Yes	☐ No
d.	CT scan	☐ Yes	☐ No

C7. For each of the following colon cancer tests, mark whether or not it usually requires people to take medicine that makes them sleepy during the test.

a.	Stool Blood Test	☐ Yes	☐ No
b.	Colonoscopy	☐ Yes	☐ No
c.	Sigmoidoscopy	☐ Yes	☐ No
d.	CT scan	☐ Yes	☐ No

C8. For each of the following colon cancer tests, if there is an abnormal test result, mark whether or not a follow-up colonoscopy is needed.

a.	Stool Blood Test	☐ Yes	☐ No
b.	Colonoscopy	☐ Yes	☐ No
c.	Sigmoidoscopy	☐ Yes	☐ No
d.	CT scan	☐ Yes	☐ No

C9. Does having a colon cancer test result that is **not** normal always mean that a person has colon cancer?

☐ Yes
☐ No

C10. How often do serious problems, such as serious bleeding or a tear in the colon, happen as a result of a colonscopy?

☐ Usually
☐ Sometimes
☐ Rarely
☐ Never

C11. For a person with an average risk for colon cancer, which test do doctors recommend be done **every year**?

☐ Stool Blood Test
☐ Colonoscopy
☐ Sigmoidoscopy
☐ CT Scan

C12. For a person with an average risk for colon cancer, which test do doctors recommend be done **every 10 years**?

- ☐ Stool Blood Test
- ☐ Colonoscopy
- ☐ Sigmoidoscopy
- ☐ CT Scan

C13. How does regular testing for colon cancer change the chances that a person will die from colon cancer?

- ☐ Increases the chance of dying from colon cancer
- ☐ Decreases the chance of dying from colon cancer
- ☐ Does not change the chance of dying from colon cancer

C14. Which colon cancer test is least likely to **miss** a cancer?

- ☐ Stool Blood Test
- ☐ Colonoscopy
- ☐ Sigmoidoscopy
- ☐ CT Scan

C15. If the results of a colon cancer test are normal, is it possible that a person could still have colon cancer?

- ☐ Yes
- ☐ No

C16. You may not know the exact number, but please take your best guess. Out of every 100 people, about how many will **die** of colon cancer? Please mark the number that you think is closest to the correct answer.

- ☐ 3
- ☐ 8
- ☐ 15
- ☐ 24
- ☐ 30
